# Gene and genetic therapies in Alzheimer’s disease and other dementias

**DOI:** 10.1016/j.tjpad.2025.100313

**Published:** 2025-07-31

**Authors:** Robert C. Alexander

**Affiliations:** Alzheimer’s Prevention Initiative, Banner Alzheimer’s Institute, United States of America

One of the tenets of drug development is that better “target validation,” i.e., a deeper understanding of the role of the drug target in the disease, predicts higher success rates in Phases 2 and 3. Genetic linkage of the target to the disease is often considered as prime evidence of target validation. In fact, a review of the causes of success and failure of AstraZeneca pipeline over a five-year period revealed that 73 % of projects with some genetic linkage of the target to the disease were active or successful in Phase 2, versus only 43 % of projects without such data [2]. More importantly, multiple gene or genetic therapies have been approved by FDA in the last 10 years for a variety of conditions with motor neuron dysfunction and/or central nervous symptoms, including spinal muscular atrophy (SMA), Duchenne’s muscular dystrophy (DMD), amyotrophic lateral sclerosis (ALS), cerebral adrenoleukodystrophy, and aromatic l-amino acid decarboxylase deficiency. So, it makes sense that gene therapy (which targets genes directly) and genetic therapy (any method that can change a gene or its expression) are now a focus in the development of therapeutics for Alzheimer’s Disease (AD) and other dementias, especially in populations where there is strong genetic linkage and high or near-certain risk of disease, such as Down’s Syndrome or autosomal dominant AD. The opportunities and challenges of applying genetic approaches to the treatment of AD and other dementias are summarized nicely in the proceedings of the EU-US CTAD Task Force, published in this issue [1].

Two of the principal challenges in the development of gene and genetic therapies for brain disorders are safety and drug biodistribution. Deaths associated with gene therapy prompted the FDA to hold a special public meeting in 2020 and since that time, fatalities have been reported following treatment with the gene therapy for SMA, ZOLGENSMA, as well as in clinical trials in DMD, Rett’s syndrome, and myotubular myopathy. These events appear to be associated with high dose, intravenous administration of adeno-associated virus (AAV) vectors. It is hoped that future improvements in capsid design (the protein shell that surrounds the viral genome), so-called “engineered” capsids, may reduce the potential for liver damage and negative immune responses.

Antisense oligomers (ASOs) have a better safety track record, but there remains a concern that the chemical modifications made to ASOs to prevent catabolism by endonucleases may cause neuroinflammation. SMA patients have a fourfold increased risk of hydrocephalus compared with non-SMA controls in the era preceding nusinersen treatment [3], and increased cerebral spinal fluid white blood cells and total protein, as well as other measures of neuroinflammation, are seen with ASOs used for different CNS diseases. Increased inflammation could exacerbate the underlying CNS disease, whether Huntington’s Disease (HD), ALS or AD, and could eventually obstruct CNS outflow and cause hydrocephalus, albeit infrequently. Despite these concerns, ASO or siRNA approaches could be used to “derisk” AAV treatments by providing proof of principle of, for example, gene silencing, with a “reversible” treatment. This assumes however that effective biodistribution of these treatments can be achieved.

Biodistribution of ASO, siRNA and AAV therapies remains a considerable hurdle. While work is rapidly advancing to develop vectors that are able to pass the blood-brain barrier, with some approaching the clinic soon, at present, drug administration for brain disorders is limited to intrathecal or intracisternal magma delivery. Intraparenchymal administration of the *APOE2* gene via an AAV is also being tested, however, it is hard to imagine that this latter method of delivery can be sufficient to effectively treat disorders like dementia that broadly affect the brain. Even with intrathecal administration of ASOs, it is difficult deliver effective doses to deeper brain structures, and the failure of tominersen in HD may be in part related to inadequate biodistribution. A promising line of research is engineering ASOs to include transferrin receptor (TfR)-binding molecules. If effective, this should greatly enhance brain biodistribution [4].

The most advanced genetic therapy for AD is the ASO from Ionis/Biogen, BIIB090, targeting the *MAPT* gene, which encodes for tau. Data from an early trial are encouraging, particularly with reported presumed beneficial effects on soluble and fibrillar tau [5]. Whether these biomarker changes result in clinical benefits is currently being tested in a large Phase 2 trial and the results are greatly anticipated. While the BIIB090 study includes symptomatic participants with either mild cognitive impairment or mild AD, ultimately the goal would be to prevent the onset of symptoms in people with either biomarker evidence of AD pathology or at near certain genetic risk. Identifying the right patients to include in future prevention trials and balancing risk/benefit will be critical. As noted in the review by Jakabek et al., gene and genetic therapies for AD and related dementias are at an early stage, but these approaches are clearly a source of hope for patients and their families.

Dr. Alexander is supported by relevant grants R01 AG058468, R01 AG055444, and R01 AG074983 from the National Institute on Aging (NIA). He is one of the leaders of the Alzheimer’s Prevention Initiative (API), which is collaborating with Eli Lilly and Roche in its ongoing and planned AD prevention trials. He is also a compensated scientific advisor to Lundbeck, Novartis, Roche, Vigil Neuro and DSMB member for ImmunoBrain.

Declaration of Generative AI and AI-assisted technologies in the writing process No AI was used in the writing process.

## CRediT authorship contribution statement

**Robert C. Alexander:** Conceptualization, Writing – original draft, Writing – review & editing.

## Declaration of competing interest

The authors declare the following financial interests/personal relationships which may be considered as potential competing interests: Robert Alexander reports a relationship with National Institute on Aging that includes: funding grants. Robert Alexander reports a relationship with Novartis Pharmaceuticals Corporation that includes: consulting or advisory. Robert Alexander reports a relationship with Roche that includes: funding grants. Robert Alexander reports a relationship with Eli Lilly and Company that includes: funding grants. Robert Alexander reports a relationship with Vigil Neuroscience Inc that includes: consulting or advisory. Robert Alexander reports a relationship with H Lundbeck AB that includes: consulting or advisory. Robert Alexander reports a relationship with ImmunoBrain that includes: consulting or advisory. If there are other authors, they declare that they have no known competing financial interests or personal relationships that could have appeared to influence the work reported in this paper.
